# Optimizing Single-Cell Long-Read Sequencing for Enhanced Isoform Detection in Pancreatic Islets

**DOI:** 10.1101/2025.04.30.651101

**Published:** 2025-05-20

**Authors:** Maria S. Hansen, Christopher J. Hill, Lori Sussel, Kristen L. Wells

**Affiliations:** Barbara Davis Center, University of Colorado Anschutz Medical Campus, Aurora CO 80045

**Keywords:** single cell long read RNA-sequencing, transcriptomics, islet biology, RNA isoforms, RNA splicing

## Abstract

Alternative splicing is an essential mechanism for generating protein diversity by producing distinct isoforms from a single gene. Dysregulation of splicing that affects pancreatic function, and immune tolerance has been linked to both type 1 and type 2 diabetes. Next-generation sequencing technologies, with their short read lengths, are limited in their ability to accurately detect splice variants. Long-read sequencing technologies offer the potential to overcome these limitations by providing full-length transcript information; however, their application in single-cell RNA sequencing has been hindered by technical challenges, including insufficient read lengths and higher error rates. Furthermore, cell types that produce high levels of a single transcript, such as islet endocrine cells, can obscure identification of lower abundance transcripts. In this study, we optimized a protocol for single-cell long-read sequencing in pancreatic islets to improve read length and transcript detection. Our findings demonstrate that 5’ library preparation protocols outperform 3’ protocols, resulting in better transcript identification. Furthermore, we show that targeted depletion of insulin transcripts enhances the detection of informative reads, highlighting the utility of transcript depletion strategies. This optimized protocol enables isoform-specific gene expression analysis and reveals differential transcript usage across the various cell types in pancreatic islets. By leveraging this approach, we gain deeper insights into the transcriptomic complexity and cellular heterogeneity within pancreatic islets.

## Introduction

Alternative splicing (AS) plays a critical role in generating protein diversity from the ~22,000 known protein-coding genes, leading to the production of over 140,000 distinct transcripts [1]. This process allows for the generation of proteins with different amino acid sequences, impacting their functions and localization within the cell, and allowing them to respond readily to changes in the environment [2, 3]. Splicing dysregulation is a key factor in many diseases, including diabetes, either due to inherent mutations in splice sites or RNA-binding proteins (RBPs), or in response to changes in environmental conditions, such as inflammatory stress or hyperglycemia [4, 5]. In the context of type 1 diabetes (T1D), diversity in isoform expression has significant implications for pancreatic function and immune tolerance. For example, differential isoform expression of autoantigens IA-2 and G6pc2 between the pancreas and thymus has been proposed to contribute to the generation of autoreactive T cells in T1D [6, 7]. Furthermore, dysregulated splicing events have been observed in islets from individuals with type 2 diabetes (T2D), underscoring the importance of splicing regulation in maintaining proper cellular function and immune homeostasis [5]. As one specific example, SNAP-25, a component of the SNARE complex responsible for vesicle fusion and exocytosis, exists as two isoforms (SNAP-25a and SNAP-25b). In SNAP-25b-deficient mice, [Ca^2+^] elevations are prematurely activated and delayed in termination, and insulin secretion is increased [8].

Despite the critical need to detect splice variants in the context of diabetes, next generation sequencing (NGS) technologies remain insufficient for this task. Identifying isoform-specific gene expression requires sequencing reads that span multiple exons of the mRNA transcript. In the human genome, transcript lengths are estimated to average between 1,800 and 4,900 bp, with the mode of the distribution around 2,000 bp [9]. NGS technologies have read lengths of 150 base pairs, making it difficult to identify isoforms. In contrast, long-read sequencing technologies, such as PacBio and Oxford Nanopore Technologies, offer the generation of full-length reads that can capture the full RNA molecule, thereby providing a clearer picture of isoform diversity. Published single-cell long-read RNA sequencing (sclrRNA-seq) libraries often report shorter read lengths than expected, which may limit transcript coverage and isoform detection. For instance, a recent study reported a median read length of 900 bp for sclrRNA-seq of two cancer cell lines [10].

Advances in sequencing technologies, especially single-cell approaches, have revealed the complex heterogeneity within the pancreas, uncovering distinct functional and transcriptomic subpopulations across different cell types. In pancreatic islets, single-cell genomics and patch-seq have identified transcriptionally and functionally distinct beta-cell subpopulations directly linking gene expression to key physiological processes such as vesicle exocytosis [11]. This heterogeneity underscores the importance of characterizing splicing events and their resulting isoforms at the single-cell level. However, sclrRNA-seq technologies come with inherent limitations. Nanopore flow cells produce fewer reads than Illumina, with around 20,000 reads per cell for a 5,000-cell experiment, well below the typical 30,000–50,000 reads per cell common in NGS. Moreover, Nanopore’s higher error rate (1%) increases the likelihood of incorrect barcode and UMI assignments. To overcome these challenges, we have optimized a protocol for pancreatic islets that improves read length, advancing the utility of long-read sequencing for single-cell transcriptomics.

### Research Design and Methods

#### Dissociation of pancreatic islets

10-week-old female C57BL/6 mice were obtained from Jackson laboratories. Pancreatic islets were isolated from mice under ketamine/xylazine/acepromazine anesthesia by collagenase delivery into the pancreas via injection into the bile duct. The collagenase-inflated pancreas was surgically removed and digested. After isolation, islets were dissociated using Accutase in a 37°C bead bath for 25–30 minutes. Single-cell suspension was filtered through a 40 mm filter and quenched in RPMI media + 10% FBS. Cells were washed again with RPMI +10% FBS and with PBS + 0.1% BSA. Single-cell suspensions were loaded into a Genomics Chromium targeting 4000 cells per sample.

#### Dissociation of spleens

Spleens were isolated from mice under ketamine/xylazine/acepromazine anesthesia. Spleens were dissociated through a 70 μm strainer in cIMDM using a 3 mL syringe plunger. Cells were washed, centrifuged, and treated with 1 mL Ammonium-Chloride-Potassium (ACK) lysis buffer for 30 seconds, followed by dilution in cIMDM and a second spin. After one additional cIMDM wash, cells were resuspended in PBS + 0.1% BSA. Single-cell suspensions were loaded into a Genomics Chromium targeting 4000 cells per sample

#### scRNA-seq library preparation and insulin depletion

Single-cell libraries were prepared using either the Chromium Next GEM Single Cell 3ʹ Kit v3.1 or the Chromium Next GEM Single Cell 5’ Kit v2 following the protocol up to and including step 2.4, stopping just before fragmentation. For the optimized libraries, the following modifications were applied to the 5’ library preparation: 1 ul 10 mM dNTP solution (Thermo Scientific FERR0191) was added to the reaction in step 1.1. The extension time was increased from 45 minutes to 2 hours in step 1.5. 1 ul 10 mM dNTP solution was added to the reaction in step 2.2 and the extension time was increased from 1 minute to 3 minutes. Insulin depletion was performed on cDNA from step 2.4 of the 10X Genomics Chromium library preparation using the DepleteX^™^ RNA Depletion Panel (Insulin) kit from Jumpcode Genomics. We followed the PacBio MAS-IsoSeq protocol (December 2022, Version 1.0) with the following modifications: During RNP Complex Formation (Step A), we used 0.9 ul Cas9 instead of 2.3 ul, and 1.6 ul Insulin Guide RNA instead of 4.0 ul Single Cell Boost Guide RNA. During Bead Cleanup (Step D), we used 50 ul (1X) AMPure XP Beads instead of 75 ul 1.5X SMRTbell Cleanup Beads.

Following insulin depletion, long-read libraries were prepared from the cDNA using Sequencing Kit V14 (Nanopore SǪK-LSK114) and the PCR Expansion (Nanopore EXP-PCA001). For 3’ libraries, the *Ligation sequencing V14 — single-cell transcriptomics with 3’ cDNA prepared using 10X Genomics on PromethION (SǪK-LSK114)* protocol was used. For 5’ libraries, the *Ligation sequencing V14 - Single-cell transcriptomics with 5’ cDNA prepared using 10X Genomics on PromethION (SǪK-LSK114)* protocol was used. Short Fragment Buffer (SFB) was used for library preparation instead of Long Fragment buffer (LFB). Library Beads (LIB) were used for the flow cell priming mix stead of Library Solution (LIS). Libraries were sequenced on R10.4.1 flow cells on either a PromethION 2 Solo (P2S) or PromethION 2 Integrated (P2i).

### Data availability

All data will be made available on GEO at time of publication.

## Results

### Evaluation of read lengths and isoform detection in published single cell long read datasets

**Figure 1:**
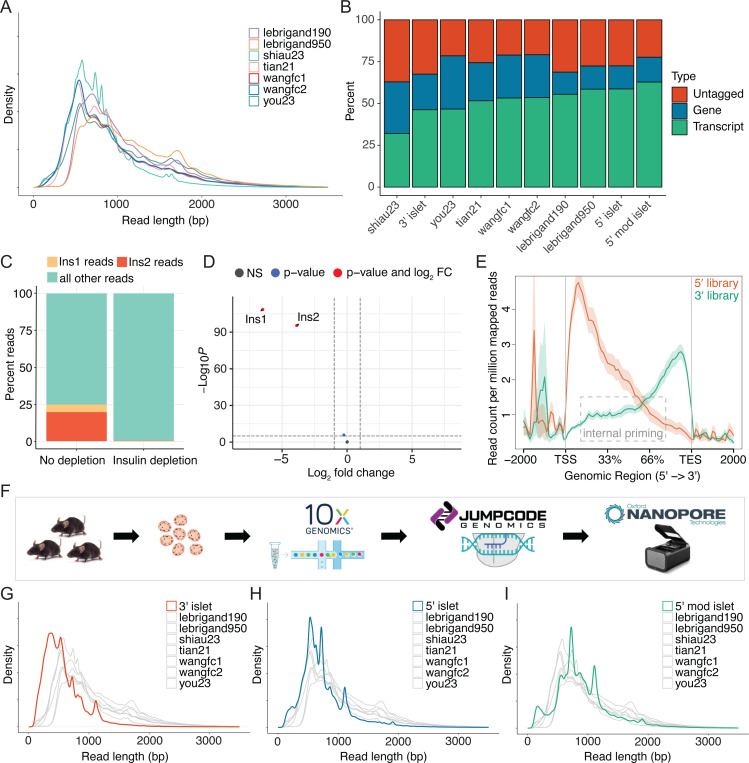
Read length and transcript identification comparison between single-cell long-read RNA-sequencing (sclrRNA-seq) libraries. (A) Read length distribution of published sclrRNA-seq libraries prepared with 10x Genomics and Nanopore technology. Biological replicates are included for datasets from Lebrigand et al., 2020 and Wang et al., 2021; other datasets are shown as single samples. (B) Proportion of reads across datasets where the gene is identified, the transcript is identified, or neither is identified. Shown are published reanalyzed datasets and three mouse pancreatic islet samples: one prepared with 3′ 10x Genomics technology, one with 5′ 10x Genomics technology, and one with 5′ 10x Genomics technology incorporating library preparation optimizations. (C) Proportion of reads aligned to Ins1 or Ins2 in a single-cell RNA-seq analysis of mouse pancreatic islets pre- and post-insulin depletion. (D) Volcano plot depicting differential gene expression between non-depleted and insulin-depleted bulk RNA-seq libraries from mouse pancreatic islets. (E) NGS coverage plot indicating read start sites across the genomic region. Libraries are single cell 10x Genomics preparations derived from human DTC melanoma cells. (F) Overview of the experimental workflow. (G) Read length distribution comparing a mouse pancreatic islet sclrRNA-seq library prepared using 3′ 10x Genomics technology to published sclrRNA-seq datasets. (H) Read length distribution comparing a mouse pancreatic islet sclrRNA-seq library prepared with 5′ 10x Genomics technology to published sclrRNA-seq datasets. (I) Read length distribution comparing a mouse islet sclrRNA-seq library prepared using 5′ 10x Genomics technology with protocol optimizations to published sclrRNA-seq datasets.

We aimed to identify isoform differences between islet cell types and subtypes using single cell RNA-sequencing. To evaluate the ability of single cell sequencing technologies to generate full-length reads, we reanalyzed previously published sclrRNA-seq datasets generated using 10x Genomics and Oxford Nanopore Technologies, focusing on their ability to capture full-length transcripts and detect isoform-specific transcript expression. Our analysis included seven sclrRNA-seq libraries from five different studies [10, 12–15]. The reanalysis revealed an average read length of 794 bp, and an average mode of 582 bp, compared to the expected mode distribution of ~2,000 bp in the human genome [9] (Figure 1A). This discrepancy between the average read length and the expected transcript length underscores the ongoing challenge of capturing full-length transcripts. This shortfall in read length is important because it limits the transcript detection ability. Where gene detection ranges from 60–75% of total reads, transcript detection ranges from 30–60% of total reads (Figure 1B). These findings highlight the limitations of current sclrRNA-seq technologies in achieving comprehensive transcript-level resolution.

### Efficient and specific depletion of insulin from islet sequencing libraries generates enhanced read diversity

Analyzing transcript expression requires a higher overall read depth than gene expression analysis, as each gene is associated with multiple transcripts. Initial analysis of our sclrRNA-seq libraries of mouse pancreatic islets led to the discovery that the two mouse insulin genes, *Ins1* and *Ins2* made up 25% of the total reads, impeding our ability to achieve optimal read depth (Figure 1C). To overcome this issue, we incorporated an insulin depletion step into the protocol and validated the specificity and efficiency of insulin depletion in a bulk short-read RNA-sequencing library of mouse pancreatic islets. The depletion was remarkably efficient and highly specific: insulin transcripts were uniquely depleted, while all other genes remained completely unaffected (Figure 1D). The same insulin depletion was then applied to a single cell pancreatic islet library followed by long-read Nanopore sequencing. Importantly, the insulin depletion was as efficient as in the bulk sample (Figure 1C). This strategy was applied to all subsequent pancreatic islet libraries generated for this study.

### Protocol modifications enhance read length and transcript identification in islet single cell long read libraries

Most high-throughput sclrRNA-seq methods rely on 10x Genomics single-cell capture and library preparation, which was originally optimized to generate and amplify shorter sequences, raising the question of whether it can effectively amplify full-length transcripts. 10x Genomics offers two types of transcriptomic profiling for single-cell RNA-seq: one that captures the 3’ end of transcripts and another that captures the 5’ end. Studies have shown that 3’ RNA libraries frequently contain internal priming artifacts [16] that would prevent the amplification of full length reads. To test for internal priming in 3’ vs 5’ libraries, we downloaded libraries generated using each technology in human melanoma samples from the datasets created by 10x genomics and analyzed the genomic coverage [17]. 3’ libraries exhibited a notably higher degree of internal priming compared to 5’ libraries, as evidenced by an increased number of reads mapping to the central regions of transcripts in genomic coverage plots (Figure 1E). Because reads generated through internal priming cannot span the full length of a transcript, this phenomenon likely contributes to the shorter read lengths observed in these libraries. To test this, sclrRNA-seq libraries were prepared from mouse pancreatic islets in parallel using both 3’ and 5’ capture technologies (Figure 1F). Remarkably, the library prepared with 5’ technology generated longer reads than the 3’ library (p < 2 × 10^−16^) (Figure 1G–H) and provided substantially improved transcript identification (Figure 1B).

To further improve the read length, several additional optimization steps were introduced into the islet 5’ library preparation protocol (Chromium Next GEM Single Cell 5’ Reagent Kits v2), including increasing the extension time from 45 minutes to 2 hours during GEM-RT Incubation and from 1 to 3 minutes during cDNA amplification, based on the approach outlined by Lebrigand et al. [12] and increasing the amount of dNTPs. Remarkably, these modifications resulted in longer reads than those from the 5’ library without modifications (p < 2 × 10^−16^) (Figure 1I), and better transcript identification than any of the published datasets (Figure 1B). Overall, this emphasizes the preference for 5’ capture over 3’ capture and highlights the necessity for library prep optimizations to enhance the amplification of full-length reads.

Isolating high-quality RNA from pancreatic islets is notoriously difficult, primarily due to the presence of digestive enzymes, including RNases that are secreted by the exocrine pancreas. To explore whether a different cell type might yield still longer reads, we applied 3’, 5’, and 5’ optimized library preparations, as described above, to lymphocytes isolated from dissociated mouse spleens. The 5’ lymphocyte sample demonstrated better transcript identification compared to the 3’ pancreatic islet sample (Supplemental figure 1). However, the 10x Genomics Chromium library prep modifications for the 5’ sample did not yield the same improvements in the lymphocyte sample as observed with the pancreatic islet sample (Supplemental figure 1). This suggests that the benefits of these optimizations might be tissue-specific and highlights the need for further refinements tailored to different tissue types.

### Isoform variants identified between alpha and beta cells and within beta cell subpopulations

**Figure 2:**
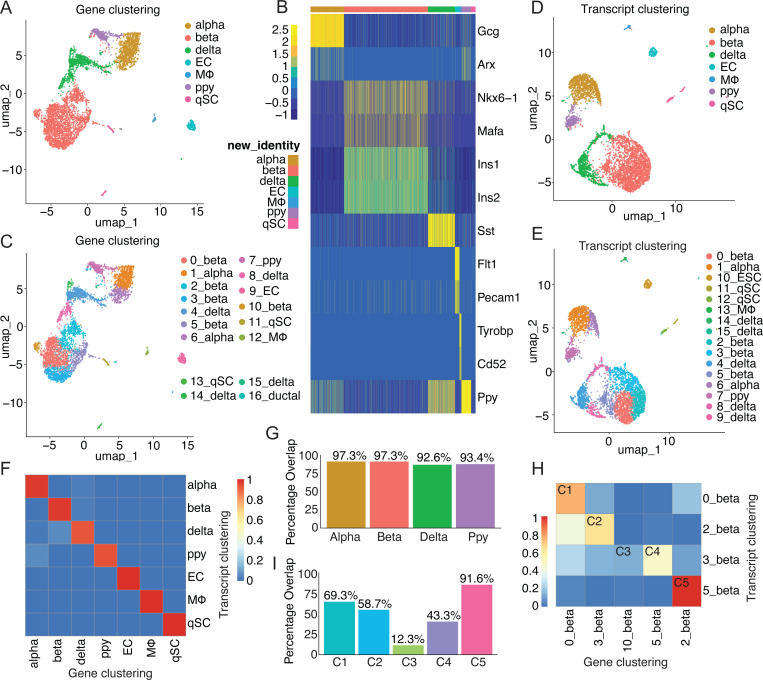
Comparison of single-cell clustering based on gene expression versus transcript-level expression. (A) UMAP projection of single cells based on gene-level expression. Cells are grouped by gene expression profiles reflecting major pancreatic cell types. (B) Heatmap showing expression of cell type-specific markers across single cells from mouse pancreatic islets. (C) UMAP projection of single cells based on gene-level expression, grouped by gene expression profiles reflecting cell subpopulations. (D) UMAP projection of single cells based on transcript-level (isoform) expression. Grouped by transcript expression profiles reflecting major pancreatic cell types. (E) UMAP projection of single cells based on transcript-level expression, grouped by transcript expression profiles reflecting cell subpopulations. (F) Confusion matrix showing concordance in cell type identification between gene-based and transcript-based clustering. (G) Bar plot quantifying cell type concordance between clustering methods. (H) Confusion matrix showing low concordance in beta cell subpopulation identification between clustering methods. (I) Bar plot quantifying beta cell subpopulation concordance between clustering methods.

With the improved library preparation, the optimized 5’ sclrRNA-seq dataset from mouse pancreatic islets was used to explore whether splicing changes could be detected from different cell types and cell states. Importantly, the sclrRNA-seq dataset allowed clear identification of all expected cell populations (Figure 2A–B). Furthermore, the analysis revealed that cell type identification remains robust whether using gene-level or transcript-level expression data for dimensionality reduction and clustering, with over 90% concordance between the two approaches (Figure 2D–G). This stability in broad cell type classification aligns with the understanding that major cell types are defined by distinct gene expression patterns. However, when examining substructure within these cell types, substantial differences emerged between gene-level and transcript-level analyses, with consistency ranging from 12% to 92% across subclusters (Figure 2I, Supplemental figure 2). These findings suggest that while gene-level expression is sufficient for identifying major cell types, transcript-level analysis provides crucial insights into subtle variations within cell populations. Such variations may reflect different cell states, functions, or responses that are not captured by gene-level analysis alone.

**Figure 3:**
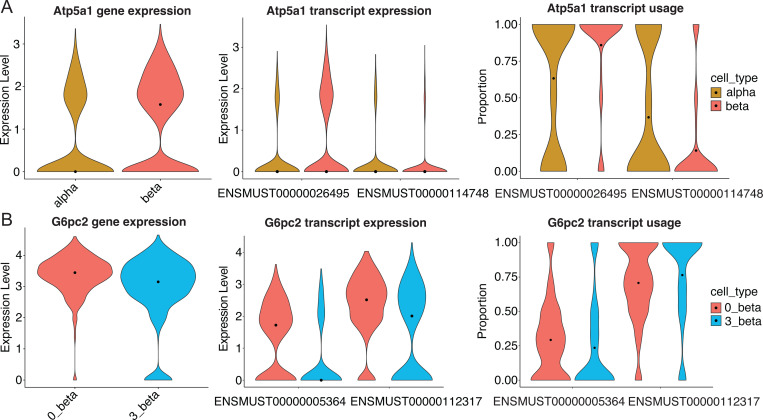
Differential transcript usage between cell types and cell subpopulations. (A) Differential transcript usage between cell types and cell subpopulations. (A) Differential gene expression (DGE), differential transcript expression (DTE), and differential transcript usage (DTU) analysis of Atp5a1. (B) DGE, DTE, DTU analysis of G6pc2.

The primary strength of sclrRNA-seq lies in its ability to capture cell-specific isoform expression. To assess differential splicing, differential transcript usage (DTU) analysis was conducted alongside differential gene expression (DGE) analysis. DTU analysis [18] identifies proportional differences in the transcript composition of a gene, comparing how much each transcript contributes to the total gene expression across conditions. Using this analysis, 342 DTU events were identified between alpha and beta cells, and 57 DTU events across subpopulations of beta cells (Supplemental table). Specifically, when comparing alpha and beta cells, we identified isoform-specific differences in Atp5a1, a gene involved in ATP production and insulin and glucagon secretion (Figure 3A). Similarly, G6pc2, a known autoantigen in T1D, displays distinct isoform expression between two beta cell subpopulations (0_beta and 3_beta), despite similar overall gene expression levels (Figure 3B). Interestingly, alternative splicing of G6pc2 has been shown to drive differential expression of *GcPC2* transcripts between the pancreas and thymus, highlighting its potential as a critical target for isoform-specific studies [7]. Neither of these genes were identified by DGE, underscoring the importance of long read sequencing for identifying previously unidentified RNA differences between cell types and cell states.

## Discussion

This study demonstrates how an improved sclrRNA-seq library preparation protocol from isolated islets produces longer reads and increases the proportion of reads that can be confidently assigned to specific transcripts, improving the utility of long-read sequencing data for identifying splice variants and cellular heterogeneity in pancreatic endocrine cell populations. Specifically, this study demonstrates that islet sclrRNA-seq libraries prepared with 5’ protocols outperform those prepared with 3’ protocols for long-read sequencing. Enhancements to the 5’ library preparation further improves read length and transcript tagging efficiency in pancreatic islets. Furthermore, depleting insulin transcripts from the pancreatic islet libraries proved to be a highly effective strategy for maximizing informative reads, demonstrating the broader potential of targeted transcript depletion in single-cell RNA-sequencing experiments.

While the modified 5’ protocol significantly improved read length in islet samples, lymphocyte samples showed significant improvement only with the unmodified 5’ protocol, with no additional benefit from the modifications. This indicates that individual cell types will require unique modifications and further optimizations. Despite the significant improvements in read length achieved with the modified protocol, it did not meet expectations for full-length transcript coverage. Achieving this goal will require further modifications to the 10x chemistry, including adjustments to the master mix and reverse transcriptase.

Although full-length coverage was not achieved for all transcripts, we successfully analyzed transcript expression and identified differential transcript usage across cell types and cell subpopulations. These advancements are critical for uncovering the full complexity of transcriptomes and hold immense potential for broad application across tissues, enabling deeper insights into cellular heterogeneity, isoform regulation, and functional diversity. Furthermore, investigating these variations at the single-cell level enables us to uncover the intricate heterogeneity within tissues, offering a deeper understanding of the functional and transcriptional diversity that would otherwise go unnoticed. Understanding splicing dysregulation in pancreatic islets is particularly important, as it may reveal how alternative splicing shapes beta cell function, immune tolerance, and beta cell susceptibility in diabetes.

## Supplementary Material

Supplement 1

Supplement 2

## Article Highlights

- This study addresses the limitations of current single-cell long-read RNA-sequencing (sclrRNA-seq) technologies in detecting full-length transcripts and isoform diversity, particularly in pancreatic islets.
- We sought to determine whether optimizing single-cell library preparation protocols could enhance read length and transcript identification in pancreatic islets.
- We found that 5’ capture methods, combined with targeted insulin depletion and extended reverse transcription, significantly improved read length and isoform detection compared to standard protocols, while maximizing the number of informative reads.
- These improvements yield longer reads in single-cell experiments, substantially enhancing transcript identification and enabling more accurate analysis of isoform diversity.
