# Supplementary material for: Optimizing Single-Cell Long-Read Sequencing for Enhanced Isoform Detection in Pancreatic Islets
Source: bioRxiv. 2025 Sep 19:2025.04.30.651101. Preprint. [Version 3] doi: 10.1101/2025.04.30.651101 (PMC12139739; doi:10.1101/2025.04.30.651101)
Supplement: 5 [file NIHPP2025.04.30.651101v3-supplement-5.pdf]

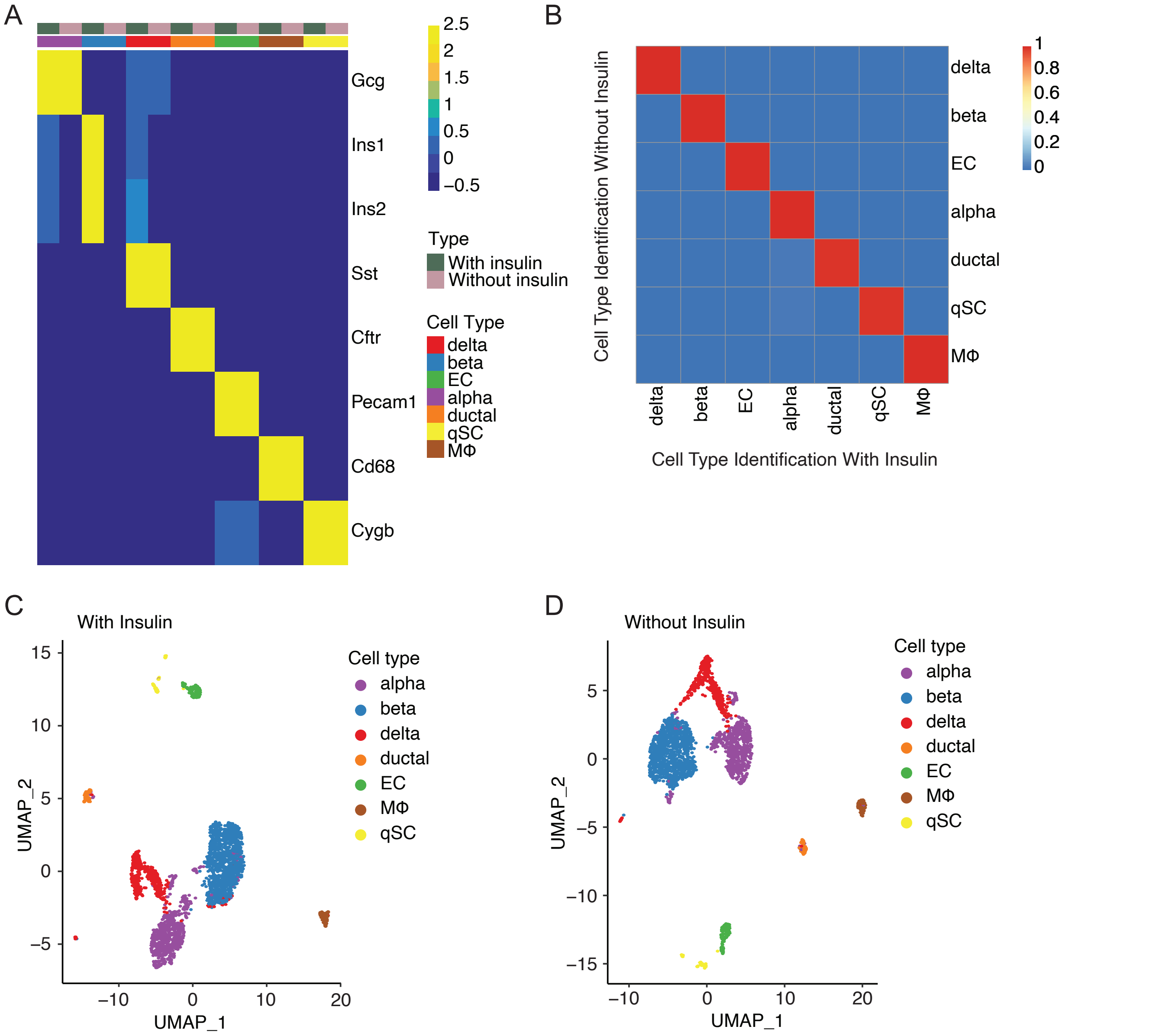

Cell type identification in mouse pancreatic islets with and without insulin transcripts. (A) Heatmap showing expression of cell type–specific markers across clusters defined by gene expression in a dataset including insulin compared to the same dataset from which insulin transcripts have been computationally removed. (B) Confusion matrix comparing cell type assignments between datasets with and without insulin transcripts. (C) UMAP projection of single cells from dataset with insulin, colored by cell type. (D) UMAP projection of single cells from dataset without insulin, colored by cell type.

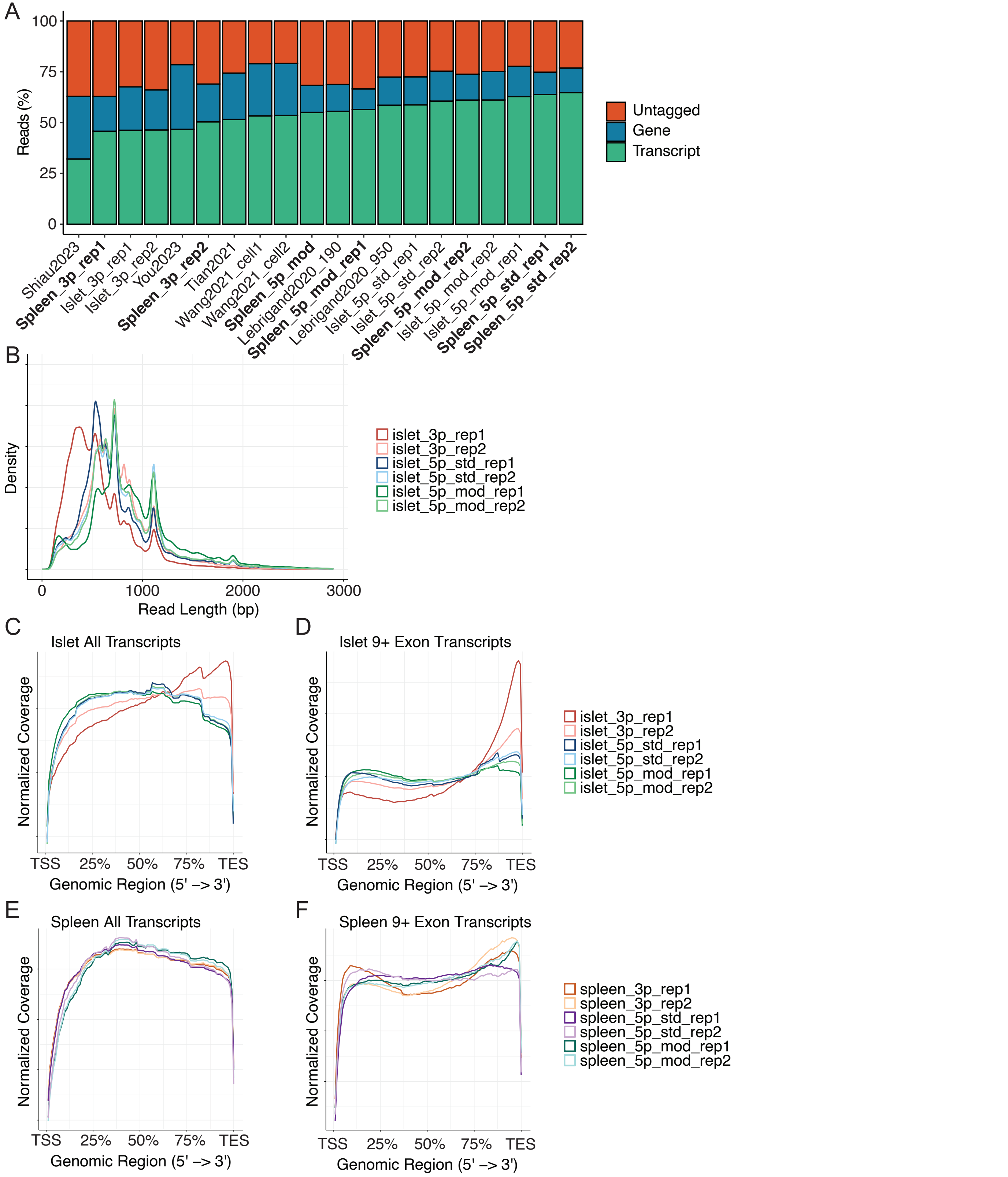

(A) Proportion of reads across datasets where the gene is identified, the transcript is identified, or neither is identified. Shown are six mouse pancreatic islet samples and six mouse spleen samples generated in this study, alongside published reanalyzed datasets. Each tissue from this study includes two biological replicates prepared with 3′ 10x Genomics technology, two with 5′ 10x Genomics technology, and two with 5′ modified 10x Genomics technology. (B) Read length distribution of mouse pancreatic islet scRNA-seq libraries, separated by replicate. Includes two replicates prepared with 3′ 10x Genomics technology, two replicates with 5′ 10x Genomics technology, and two replicates with 5′ 10x Genomics technology with library preparation optimizations. (C–F) Coverage plots showing Softmax-normalized read coverage across relative transcript positions in mouse pancreatic islet (C, D) and spleen (E, F) scRNA-seq libraries prepared with 3′, 5′, or 5′ modified 10x Genomics technology. Samples separated by replicate. Panels C and E include all multi-exon transcripts, while panels D and F include only transcripts with ≥9 exons.

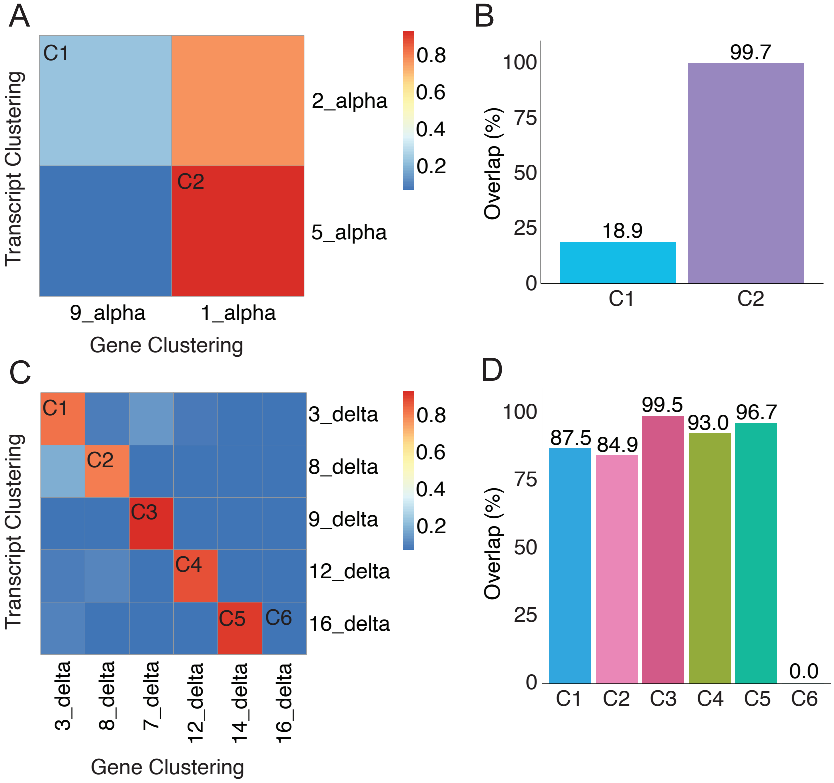

(A) Confusion matrix showing concordance in alpha cell subpopulation identification between gene-based and transcript-based clustering. (B) Bar plot showing concordance of alpha cell subpopulations across clustering methods. Concordance is quantified for two comparisons: C1 (comparison 1) and C2 (comparison 2). (C) Confusion matrix showing low concordance in delta cell subpopulation identification between clustering methods. (D) Bar plot quantifying delta cell subpopulation concordance between clustering methods quantified for comparisons C1-C6.

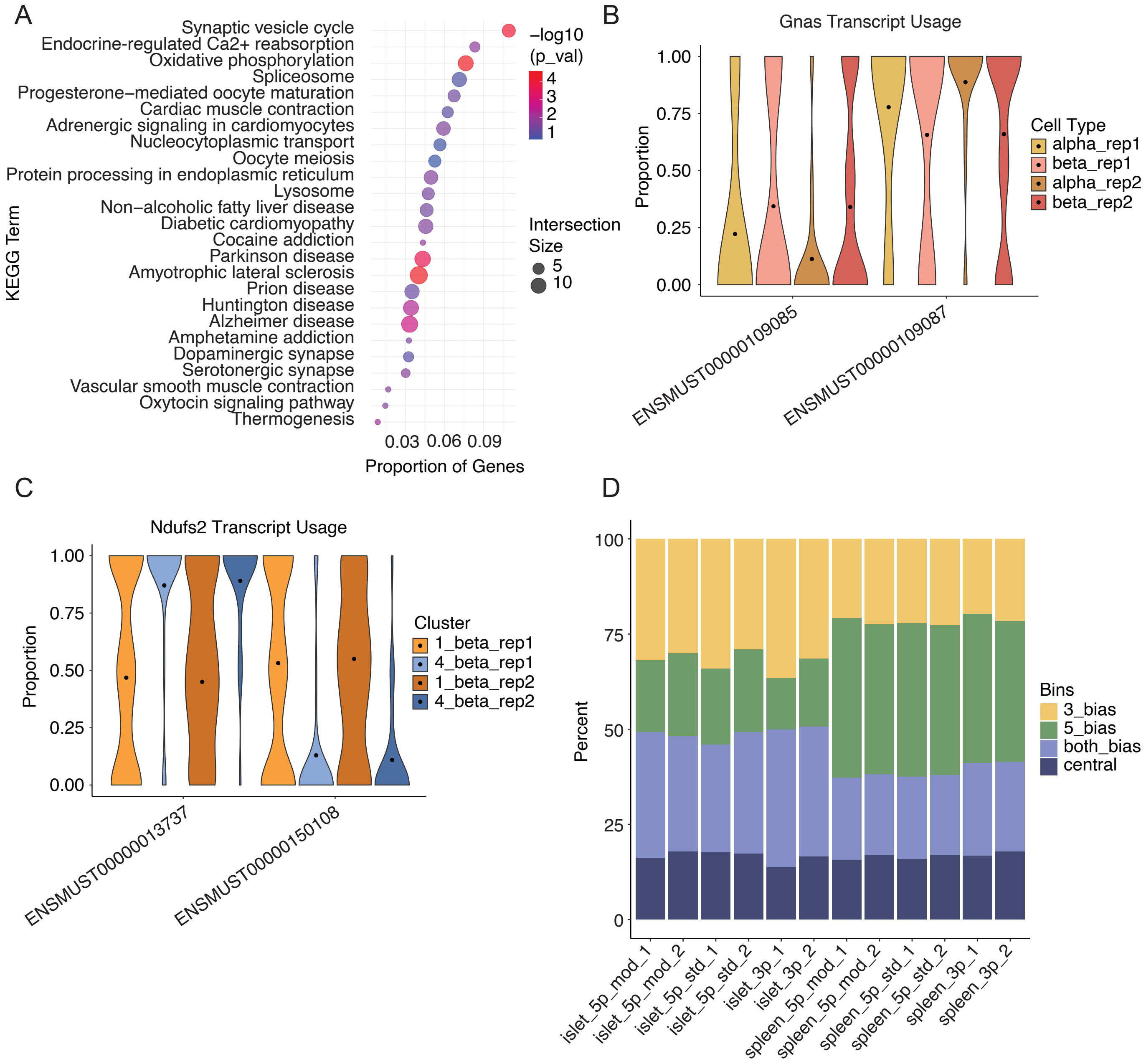

(A) KEGG pathway enrichment for DTU genes between beta cell subpopulations 1_beta and 4_beta. The x-axis shows the proportion of functionally annotated genes in the query that are associated with each pathway. Bubble size represents the number of DTU genes in the pathway, and bubble color indicates statistical significance (-log10 p-value). The top 25 enriched KEGG pathways are shown. (B) Differential transcript usage (DTU) analysis of Gnas between alpha and beta cells, separated by biological replicate. (C) DTU analysis of Ndufs2 between beta cell subpopulations 1_beta and 4_beta, separated by biological replicate. (D) Bar plot showing 3′ versus 5′ bias across libraries prepared with different technologies. Transcripts are classified by whether the junctions that uniquely identify each isoform fall in the 3′ region or 5′ region of the transcript, or both.

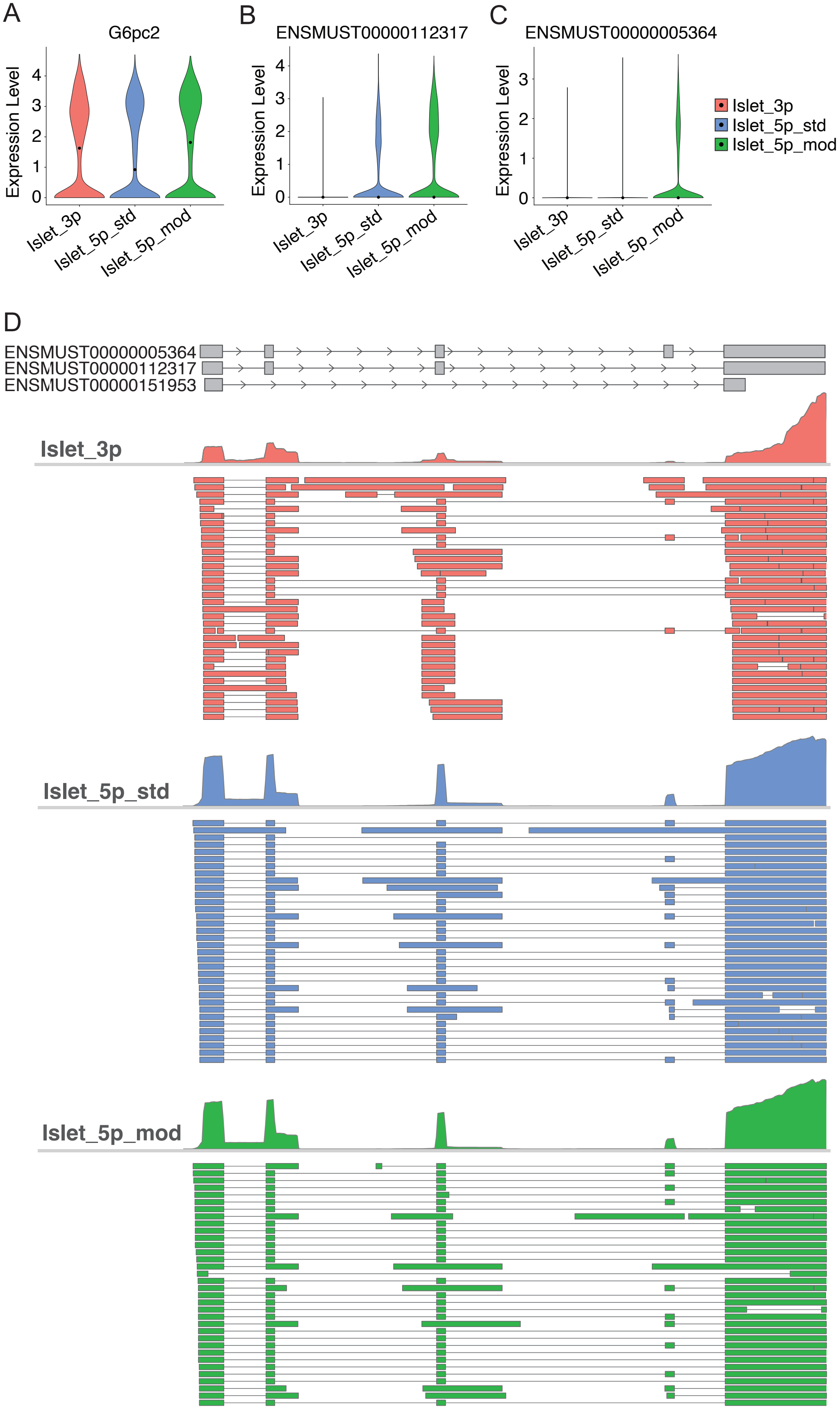

G6pc2 gene and transcript expression across mouse pancreatic islet libraries. (A) Gene-level expression of G6pc2 in 3′ modified, 5′ standard, and 5′ modified libraries (two biological replicates merged for each library). (B) Transcript-level expression of ENSMUST00000112317, isoform of G6pc2, in the same libraries. (C) Transcript-level expression of ENSMUST00000005364, isoform of G6pc2, in the same libraries. (D) Gviz plot showing a gene model track of select transcripts of G6pc2, and an alignments track including all reads uniquely aligned to any transcript of G6pc2.

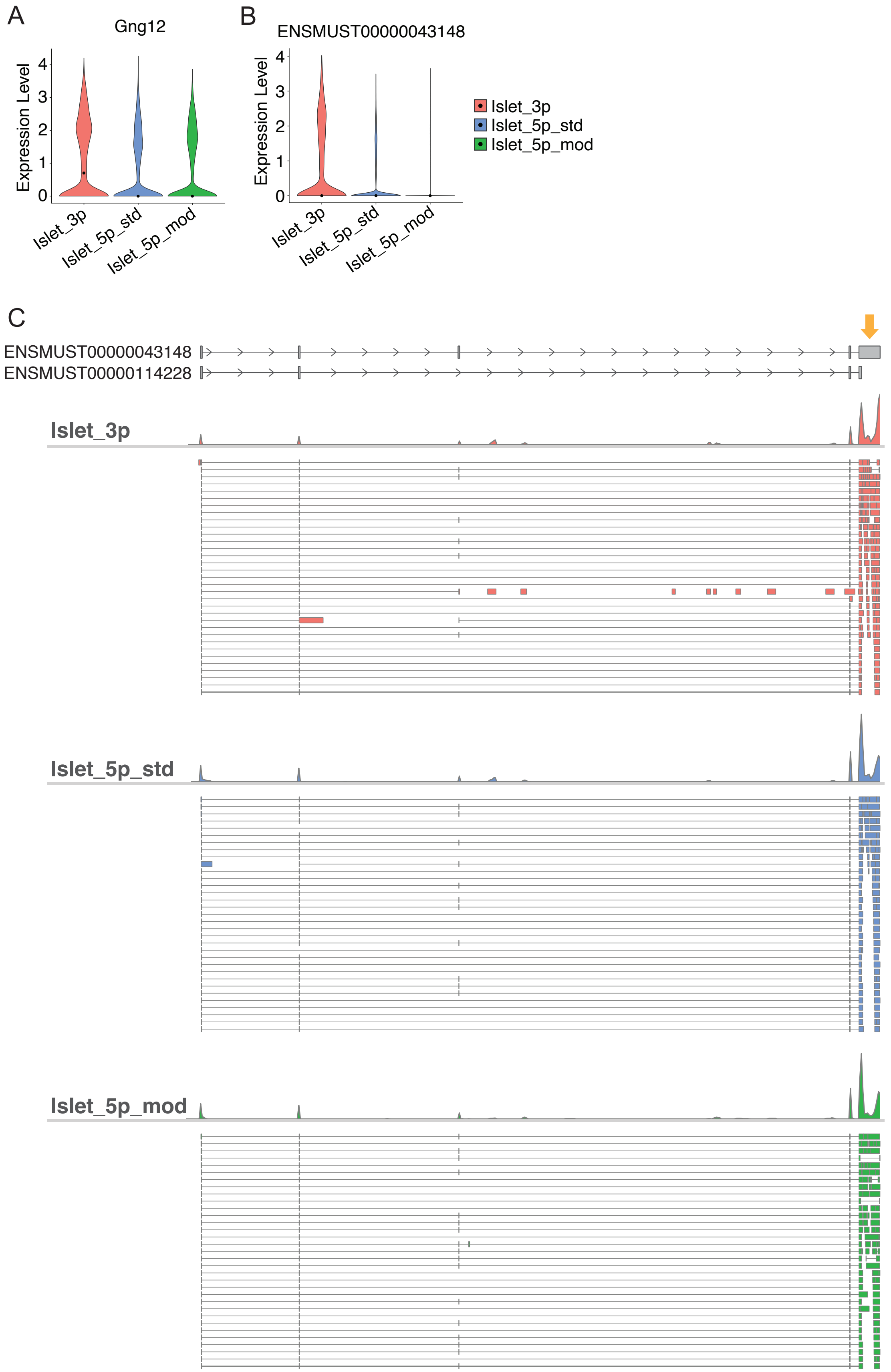

Gng12 gene and transcript expression across mouse pancreatic islet libraries. (A) Gene-level expression of Gng12 in 3′ modified, 5′ standard, and 5′ modified libraries (two biological replicates merged for each library). (B) Transcript-level expression of ENSMUST00000043148, isoform of Gng12, in the same libraries. (C) Gviz plot showing a gene model track of select transcripts of Gng12, and an alignments track including all reads uniquely aligned to any transcript of Gng12. Arrow indicates a long exon at the 3’ end of the transcript.

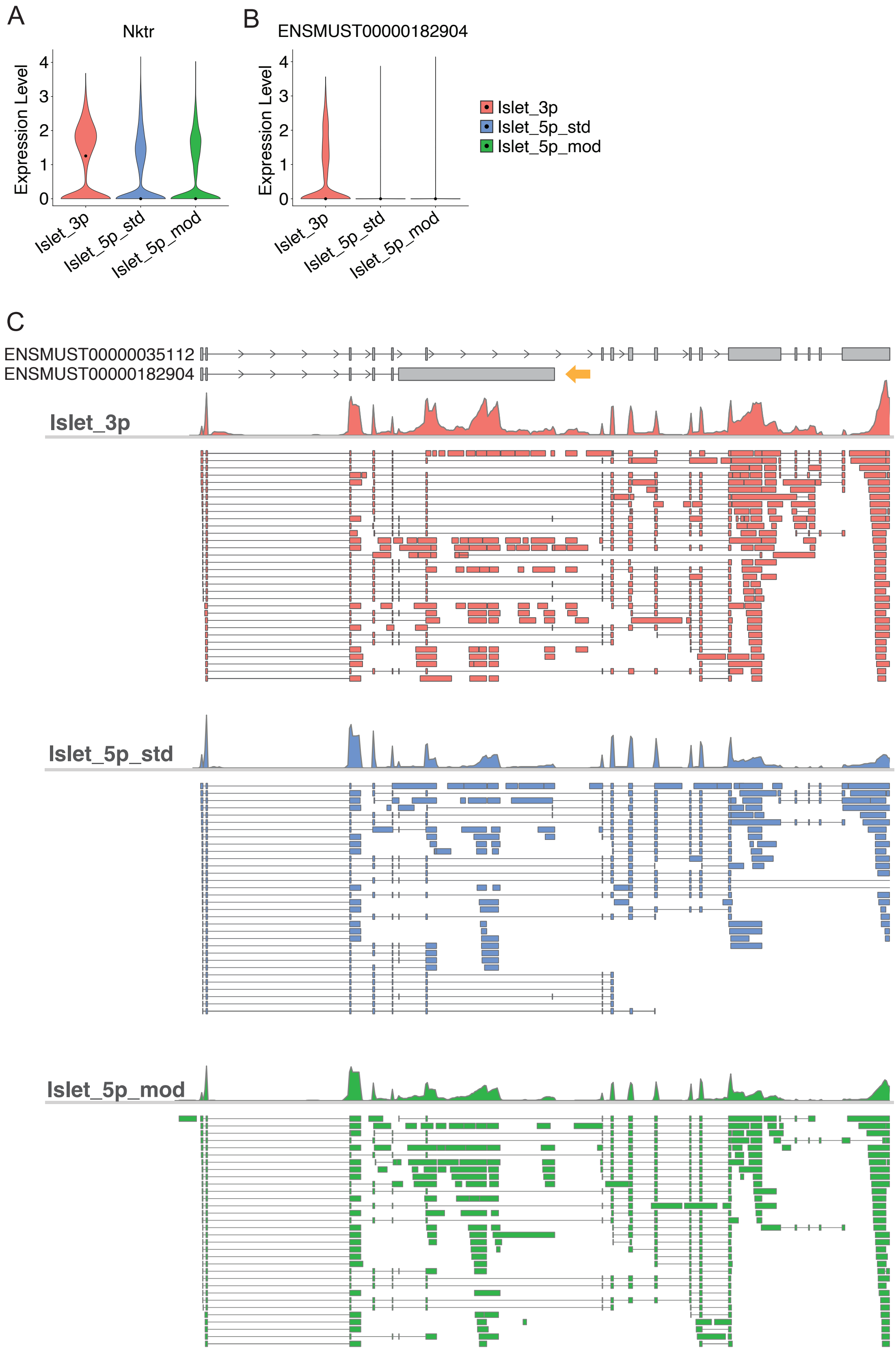

Nktr gene and transcript expression across mouse pancreatic islet libraries. (A) Gene-level expression of Nktr in 3′ modified, 5′ standard, and 5′ modified libraries (two biological replicates merged for each library). Transcript-level expression of ENSMUST00000182904, isoform of Nktr, in the same libraries. (C) Gviz plot showing a gene model track of select transcripts of Nktr, and an alignments track including all reads uniquely aligned to any transcript of Nktr. Arrow indicates retained intron captured by internal priming.
