# Supplementary material for: Optimizing Single-Cell Long-Read Sequencing for Enhanced Isoform Detection in Pancreatic Islets
Source: bioRxiv. 2025 May 20:2025.04.30.651101. Preprint. [Version 2] doi: 10.1101/2025.04.30.651101 (PMC12139739; doi:10.1101/2025.04.30.651101)
Supplement: Supplementary file 1 [file NIHPP2025.04.30.651101v2-supplement-2.pdf]

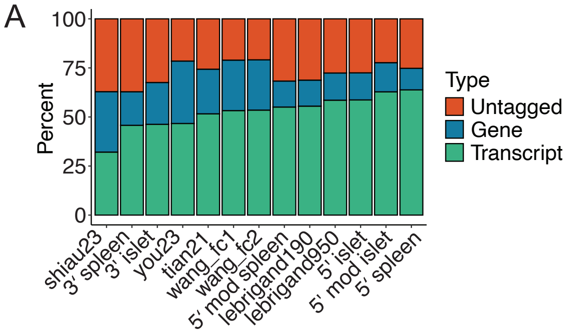

Supplemental Figure 1: (A) Proportion of reads across datasets where the gene is identified, the transcript is identified, or neither is identified. Shown are reanalyzed published datasets, three mouse pancreatic islet samples (prepared with 3′ 10x Genomics technology, 5′ 10x Genomics technology, and 5′ 10x Genomics technology with library preparation optimizations), and three mouse lymphocyte/spleen samples prepared using the same methods as the pancreatic islet libraries.technology, and 5′ 10x Genomics technology with library preparation optimizations), and three mouse spleen samples prepared using the same methods as the pancreatic islet libraries.

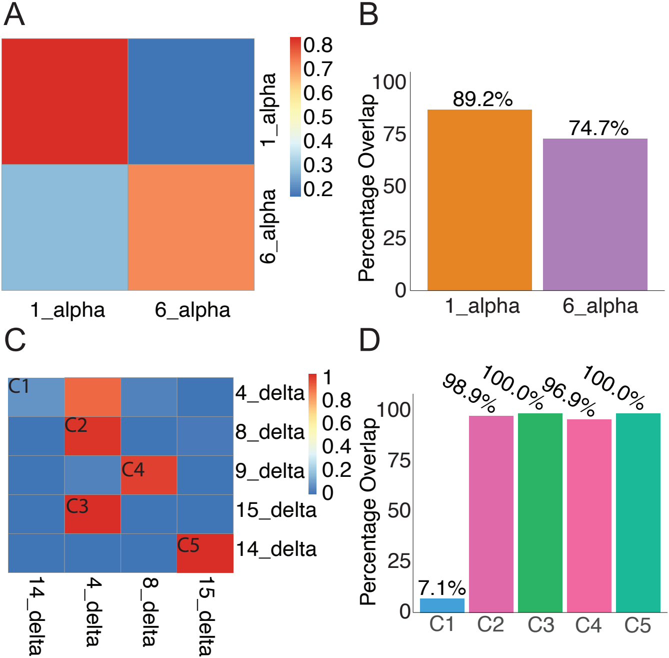

Supplemental Figure 2: Confusion matrix showing concordance in alpha cell subpopulation identification between gene-based and transcript-based clustering. (B) Bar plot quantifying alpha cell subpopulation concordance between clustering methods. (C) Confusion matrix showing low concordance in delta cell subpopulation identification between clustering methods. (D) Bar plot quantifying delta cell subpopulation concordance between clustering methods.
