# Supplementary material for: Optimizing Single-Cell Long-Read Sequencing for Enhanced Isoform Detection in Pancreatic Islets
Source: bioRxiv. 2025 May 20:2025.04.30.651101. Preprint. [Version 2] doi: 10.1101/2025.04.30.651101 (PMC12139739; doi:10.1101/2025.04.30.651101)
Supplement: Supplementary file 2 [file media-1.pdf]

Supplemental methods

Bulk RNA-seq library preparation

Islets were isolated from two male C57BL/6J mice as described in the main methods. RNA was purified using the Qiagen RNeasy micro kit (Qiagen Cat # 74004). Bulk RNA-seq libraries were prepared using KAPA mRNA Hyper Prep kit (Roche 8098123702), following the KAPA mRNA HyperPrep Kit protocol (KR1352 – v7.21). Insulin depletion was applied between step 8 and 9 in the KAPA library prep protocol.

Single cell RNA-seq preprocessing

Single cell libraries were processed using the EPI2ME single cell workflow v1.1.0 from Oxford Nanopore Technologies for the identification of cell and UMI barcodes (https://github.com/epi2me-labs/wf-single-cell) using the mm10-2020-A mouse reference provided by 10x Genomics. The kit name was either 3prime or 5prime depending on the 10x library preparation and we provided the kit version v3 and v1 for the 3' and 5' samples we prepared respectively. We provided the kit version that was indicated in the publications associated with each published dataset. All parameters can be found on github (https://github.com/CUAnschutzBDC/maria_long_read_081224/blob/61d52b6ef94ff974a394a9335dd65d28b9e6360e/01_wf_single_cell/trial_parameters.tsv) The pipeline was altered to bypass the StringTie step to use a consistent GTF file from the mm10 genome. These jobs were executed using Singularity and Nextflow. Only the reads that had a sequence quality higher than 7 were input into this pipeline.

We manually created a gene count matrix from the BAM outputs that were produced by the EPI2ME pipeline, then utilized the emptyDrops function from the DropletUtils package (Lun, Riesenfeld et al. 2019) to identify cell barcodes. Using these barcodes, we subset the gene and transcript count matrices from the single cell workflow and calculated the read lengths of cells that were identified using the barcodes. Read lengths were calculated from the BAM files by looking at the length of the query sequences for all primary reads and were subset further by matching barcodes (CB), whether the read was tagged with a gene (GN), and whether the read was tagged with a transcript (TR). Finally, we examined the BAM files and calculated the number of reads that were tagged with a gene and a transcript, before creating the final output plots.

Detailed analysis of the 5' modified islet dataset

The filtered data for the 5' modified islet dataset was input into Seurat v4.1.3 (Satija, Farrell et al. 2015, Butler, Hoffman et al. 2018, Stuart, Butler et al. 2019, Hao, Hao et al. 2021, Hao, Stuart et al. 2024), using R v4.2.3. Quality control metrics were calculated using the perCellQCMetrics function from the Scuttle package (v1.8.4) (McCarthy, Campbell et al. 2017). We used the DoubletFinder package (v2.0.3) (McGinnis, Murrow et al. 2019) to remove cells that were identified as doublets, before running a PCA. We tested out several resolutions of UMAP before generating final clustering.

Cell types were initially identified using a reference dataset (Baron, Veres et al. 2016) and further refined based on the expression of known islet cell type markers. Dimensionality reduction and clustering was first performed using gene-level expression data, followed by

a second round of clustering based on transcript-level expression. To evaluate the consistency between gene- and transcript-based cell type annotations, we calculated concordance between the two methods and visualized the results as confusion matrices using the Matrix (v1.5-4) (Jagan 2024) and pheatmap (v1.0.12) packages. Clusters identified by transcript-level expression were used for downstream analysis. To assess differential splicing, we conducted differential transcript usage (DTU) analysis using DTUrtle (v1.0.2) (Tekath and Dugas 2021) alongside differential gene expression (DGE) analysis. DTU analysis identifies proportional differences in the transcript composition of a gene, comparing the relative contribution of individual transcripts to the overall expression of a gene across conditions. DTU analysis was performed to compare alpha and beta cells, as well as to compare beta cell subpopulations.

Most figures were generated using the dplyr (v1.1.0), ggplot2 (v3.4.1), readr (v2.1.4), tidyverse (v2.0.0), MetBrewer (v0.2.0), and scAnalysisR (v0.0.0.9000) (Wells, Miller et al. 2020) packages.

Further details of this pipeline can be found in our three-step pipeline at our code repository: https://github.com/CUAnschutzBDC/maria_long_read_081224/

Docker images can be found here:

RNA-seq preprocessing (STAR, Fastqc, cutadapt, featurecounts):

https://hub.docker.com/r/kwellswrasman/rnaseq_general v2

scRNA-seq (DTurtle, scAnalysisR, Seurat):

https://hub.docker.com/r/kwellswrasman/sclrseq_methods_r_docker v1

Bulk RNA-seq analysis

Raw reads were trimmed using cutadapt (version 4.8, python version 3.10.14) (Martin 2011) and aligned to the mouse GRCm38 genome using STAR (version 2.7.11b) (Dobin, Davis et al. 2013). Gene expression was quantified with featureCounts (version 2.0.6) (Liao, Smyth et al. 2014) and differential expression analysis was conducted using DESeq2 (version 1.44.0) (Love, Huber et al. 2014) with R version 4.4.1. All plots were made using ggplot2 (version 3.5.1) in R.

NGS coverage

The 10k Human DTC Melanoma, Chromium GEM-X Single Cell 5' and 10k Human DTC Melanoma, Chromium GEM-X Single Cell 3' aligned bam files were downloaded from 10x genomics (https://www.10xgenomics.com/datasets). Coverage plots were generated using NGS plot (v2.63) (Shen, Shao et al. 2014).

References

Baron, M., A. Veres, S. L. Wolock, A. L. Faust, R. Gaujoux, A. Vetere, J. H. Ryu, B. K. Wagner, S. S. Shen-Orr, A. M. Klein, D. A. Melton and I. Yanai (2016). "A Single-Cell Transcriptomic Map of the Human and Mouse Pancreas Reveals Inter- and Intra-cell Population Structure." Cell Syst **3**(4): 346-360 e344.

Butler, A., P. Hoffman, P. Smibert, E. Papalexi and R. Satija (2018). "Integrating single-cell transcriptomic data across different conditions, technologies, and species." Nat Biotechnol **36**(5): 411-420.

Dobin, A., C. A. Davis, F. Schlesinger, J. Drenkow, C. Zaleski, S. Jha, P. Batut, M. Chaisson and T. R. Gingeras (2013). "STAR: ultrafast universal RNA-seq aligner." Bioinformatics **29**(1): 15-21.

Hao, Y., S. Hao, E. Andersen-Nissen, W. M. Mauck, 3rd, S. Zheng, A. Butler, M. J. Lee, A. J. Wilk, C. Darby, M. Zager, P. Hoffman, M. Stoeckius, E. Papalexi, E. P. Mimitou, J. Jain, A. Srivastava, T. Stuart, L. M. Fleming, B. Yeung, A. J. Rogers, J. M. McElrath, C. A. Blish, R. Gottardo, P. Smibert and R. Satija (2021). "Integrated analysis of multimodal single-cell data." Cell **184**(13): 3573-3587 e3529.

Hao, Y., T. Stuart, M. H. Kowalski, S. Choudhary, P. Hoffman, A. Hartman, A. Srivastava, G. Molla, S. Madad, C. Fernandez-Granda and R. Satija (2024). "Dictionary learning for integrative, multimodal and scalable single-cell analysis." Nat Biotechnol **42**(2): 293-304.

Jagan, D. B. a. M. M. a. M. (2024). "Matrix: Sparse and Dense Matrix Classes and Methods." from https://CRAN.R-project.org/package=Matrix.

Liao, Y., G. K. Smyth and W. Shi (2014). "featureCounts: an efficient general purpose program for assigning sequence reads to genomic features." Bioinformatics **30**(7): 923-930.

Love, M. I., W. Huber and S. Anders (2014). "Moderated estimation of fold change and dispersion for RNA-seq data with DESeq2." Genome Biol **15**(12): 550.

Lun, A. T. L., S. Riesenfeld, T. Andrews, T. P. Dao, T. Gomes, J. participants in the 1st Human Cell Atlas and J. C. Marioni (2019). "EmptyDrops: distinguishing cells from empty droplets in droplet-based single-cell RNA sequencing data." Genome Biol **20**(1): 63.

Martin, M. (2011). "Cutadapt removes adapter sequences from high-throughput sequencing reads." EMBnet. journal **17**(1): 10-12.

McCarthy, D. J., K. R. Campbell, A. T. Lun and Q. F. Wills (2017). "Scater: pre-processing, quality control, normalization and visualization of single-cell RNA-seq data in R." Bioinformatics **33**(8): 1179-1186.

McGinnis, C. S., L. M. Murrow and Z. J. Gartner (2019). "DoubletFinder: Doublet Detection in Single-Cell RNA Sequencing Data Using Artificial Nearest Neighbors." Cell Syst **8**(4): 329-337 e324.

Satija, R., J. A. Farrell, D. Gennert, A. F. Schier and A. Regev (2015). "Spatial reconstruction of single-cell gene expression data." Nat Biotechnol **33**(5): 495-502.

Shen, L., N. Shao, X. Liu and E. Nestler (2014). "ngs.plot: Quick mining and visualization of next-generation sequencing data by integrating genomic databases." BMC Genomics **15**: 284.

Stuart, T., A. Butler, P. Hoffman, C. Hafemeister, E. Papalexi, W. M. Mauck, 3rd, Y. Hao, M. Stoeckius, P. Smibert and R. Satija (2019). "Comprehensive Integration of Single-Cell Data." Cell **177**(7): 1888-1902 e1821.

Tekath, T. and M. Dugas (2021). "Differential transcript usage analysis of bulk and single-cell RNA-seq data with DTUrtle." Bioinformatics **37**(21): 3781-3787.

Wells, K. L., C. N. Miller, A. R. Gschwind, W. Wei, J. D. Phipps, M. S. Anderson and L. M. Steinmetz (2020). "Combined transient ablation and single-cell RNA-sequencing reveals the development of medullary thymic epithelial cells." Elife **9**.
